# COVID-19 in Ghana-Using the health system framework to describe RISE-mitigation strategies for COVID-19 response and recovery in Ghana

**DOI:** 10.3389/fpubh.2026.1810964

**Published:** 2026-05-25

**Authors:** Paa Kobina Forson, Doreenda Enyonam Ahiataku, Richard Owusu, Mohammed Aminu Andrew Musah, Aisha Mustapha, Stanford Adesons, Joseph Ayisah Eyeson, Albert Wuddah-Martey, Belinda Afriyie Nimako, Leah Greenspan, Erin Sullivan, John Eleeza, Franklin Asiedu-Bekoe, Kwame Amponsa-Achiano, Lawrence Ofori-Boadu, Karen Cadwell, Aimee Ogunro, Michelle Schaan, Nichodemus Gebe, Ebo Hammond, Molly Strachan, Pearl Nanka-Bruce, Zohra Balsara

**Affiliations:** 1 Jhpiego, Accra, Ghana; 2 Independent Consultant, Accra, Ghana; 3 Ghana Ministry of Health, Accra, Ghana; 4 Ghana Health Service, Accra, Ghana; 5 Jhpiego Corporation, Baltimore, MD, United States

**Keywords:** capacity building, COVID-19, Ghana, health financing, health information systems and data integration, health management and leadership, health system building blocks, Reaching Impact Saturation and Epidemic Control (RISE)

## Abstract

Navigating global crises like the Coronavirus Disease 2019 (COVID-19) demands strategic and impactful health interventions. Evaluating these interventions is crucial for fortifying health systems at both national and global scales. This article described and appraised projects carried out under the United States Government-funded, Reaching Impact Saturation and Epidemic Control (RISE) initiative during the pandemic in Ghana, offering insights and lessons learned through six health system building blocks. A mixed study design using qualitative and quantitative approaches: comprehensive document review, engagement with project managers and case narratives of data collections were adopted to appraise 10 novel interventions. Projects were selected using a census method, which included all projects that had been completed at the time of appraisal. Following selection, each project was mapped to the six building components using well-defined criteria; sustainable health financing, service provision, health management and leadership, products and logistics, information systems and data integration, and human resource. Overall, significant improvements in service delivery and health system strength were noted. COVID-19 immunization was successfully integrated into routine service delivery, resulting in 93.2% coverage attainment. Data quality audits and saturation analyses result in the institutionalization of standardized reporting and real-time data utilization. Under the Test-2-Treat (T2T) project, access to COVID-19 medications was provided to 79% confirmed cases. Additionally, RISE-supported oxygen interventions (LOX/PSA systems) improved equitable access to medical oxygen, reducing travel distances for peripheral facilities by 12.1% to 69.7% to procure medical oxygen. Capacity-building activities further strengthened service delivery and equipment maintenance, while generating critical lessons for the Ghana Health Service on integrating pandemic response interventions into routine health system functions.

## Background

What was once thought to be a regular cold with an unknown cause, Coronavirus Disease 2019 (COVID-19), spread throughout every continent in less than two years (1). Ghana confirmed its first case on March 12, 2020 (2). All public meetings were prohibited in order to detect control and prevent the spread. To identify, isolate and treat all confirmed cases, surveillance techniques, such as active case search and contact tracing were implemented throughout the lockdown (2). The pandemic unearthed weaknesses in global and national health systems (3–7), introducing a renewed focus on collaboration and coordination among government and agencies (8, 9). The response to the pandemic by countries was varied and support from international donor agencies were multifaceted. It is therefore important to take stock of all responses and interventions as a step in preparing for future pandemic. In Ghana, the Presidency, the Ministry of Health (MoH), its agency Ghana Health Service (GHS) and the Ministry of Information were central to the response to COVID-19. A comprehensive appraisal of these responses and health system interventions in the premise of global frameworks is now crucial than ever, as the world has evolved since the COVID-19 pandemic. By viewing the global health system through this framework, we can gain deeper insights and drive meaningful reforms, while learning valuable lessons from both the successes and failures (10). These framework provide a clear set of capacities and goals that can be used to study and appraise the global health system, helping to identify strengths, gaps and potential areas for reform (11). Through studies, we gain valuable insights into how different health systems operate and evolve (12–15). By leveraging on this framework, we can uncover key areas for improvement and innovation, paving the way for stronger, more resilient health systems worldwide. While various frameworks have been used to evaluate health systems across various contexts, their application in assessing interventions during the COVID-19 crisis in Ghana remains unexplored. This article appraised and illustrated the various projects the Reaching Impact, Saturation, and Epidemic Control (RISE) undertook during the COVID-19 pandemic through the lens of the health systems strengthening pillars framework.

### The global RISE initiative

Globally, the RISE project, funded by the United States Government (USG) has provided technical support, service delivery, research and comprehensive health system assistance to nearly 25 countries. Drawing on strategies proven effective for decades, RISE tackles key Human Immunodeficiency Virus (HIV) and COVID-19 challenges while aligning with the objectives of the Global Health Security Agenda. This multi-year project aims to address critical health priorities and enhance epidemic control efforts on a global scale. RISE stands out for its innovative approach, employing tailored strategies that adapt to the unique challenges faced by countries during the pandemic. Through sustained engagement with Ministries of Health and diverse local stakeholders, the initiative ensured that technical assistance was both contextually grounded and strategically aligned to address clearly defined system-level gaps within each setting.

### RISE response strategies in Ghana

**Figure 1:**
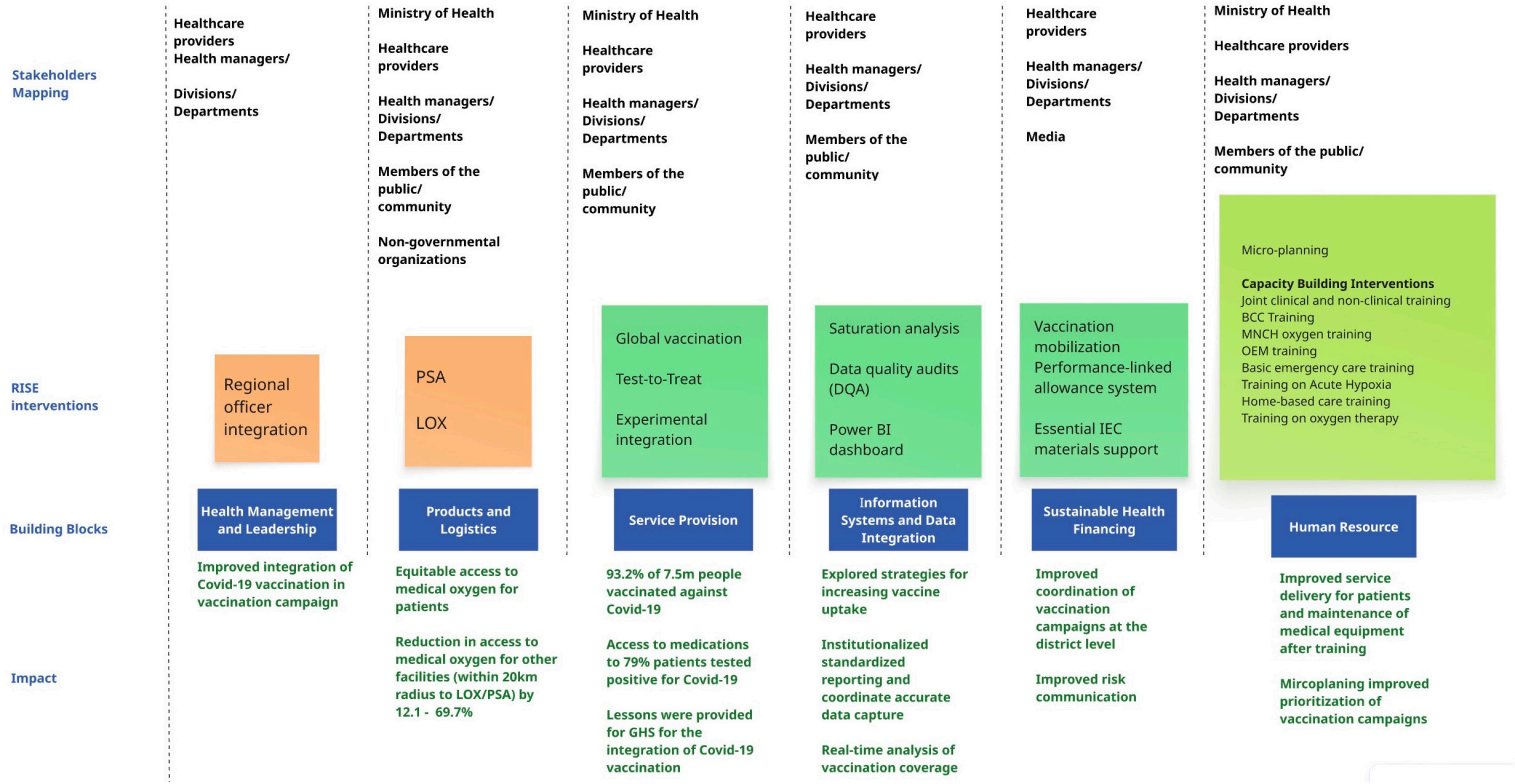
RISE-led projects.

In Ghana, working in collaboration with the Ministry of Health (MOH), Ghana Health Service (GHS), and other local and global stakeholders, the RISE project has responded and supported COVID-19 mitigating efforts through thirteen relevant strategies (Figure 1). In this article, we appraised these strategies based on these major themes – Sustainable Health System Financing, Service Provision, Health Management and Leadership, Products and Logistics, Information Systems and Data Integration and Human Resource.

## Methodology

Over the years, RISE implementation strategies have been anchored and integrated into Ghana Health Service's system strengthening initiatives. The design, development and implementation of various strategies have adopted a joint involvement of relevant partners with unique roles (Figure 1). To critically appraise these strategies, we adopted a mixed method design. Projects were selected using a census approach and were completed at the time of appraisal. After selection, all projects were mapped into the six building blocks using clearly defined criteria (Supplementary Table 1). For each project, data for appraisal was collected retrospectively through a comprehensive document review of project workplans, implementation reports and monitoring and evaluation reports. Project reports provided implementation strategies, challenges and stakeholder engagement metrics. Monitoring and evaluation reports provided quantitative key performance metrics where available, pre-and-post intervention outcomes. Qualitative data was gathered through engagement with project managers and case narratives were collected for appraisal.

### Information systems and data integration

Reliable data is key to decision-making across the health system's building blocks (11). Amidst the challenges posed by the pandemic, data was pivotal in making informed decisions. In Ghana, the Ministry of Health set an ambitious goal to vaccinate 72% of the total population against COVID-19 by the close of 2022. Yet, as of December 31, 2023, despite collaborative efforts from multiple partners, only 56.7% of the target population was fully vaccinated. In response to this shortfall, RISE implemented a *saturation analysis* survey aimed at exploring strategies to improve COVID-19 vaccination rates. The survey was conducted in 27,222 households using the Expanded Program on Immunization (EPI) cluster sampling to select eligible household individuals for interviews. Data was collected by GHS health information officers and RISE. The survey revealed that, 76.7% individuals received at least one vaccine, while 20.8% refused uptake. We also found that community members preferred the uptake of vaccines in the early hours of Sunday. Based on these key findings RISE in collaboration with GHS integrated vaccination reporting into a *Power BI dashboard*, allowing real-time analysis of vaccination coverage by districts and regions. This intervention provided crucial information necessary to refine vaccination campaigns, allowing stakeholders to geographically pinpoint areas with low and high coverage and purposefully allocate resources to optimize vaccination rates. This strategy has contributed significantly to the increase in target attainment from 56.7% to 93%. This initiative aligns with other African initiatives, as reported by Mboussou and colleagues, which implemented a quantitative tracking system to monitor COVID-19 vaccination coverage across 23 African countries. Their findings highlighted that the vaccine rollout prompted unprecedented investments in health data systems.

To plan and execute well-targeted vaccination campaigns, governments urgently needed accurate and timely vaccination data, including confirmed cases, deaths and vaccination status (16). Ghana faced challenges in promoting online data capture for COVID-19 vaccination, resulting in some health facilities using informal, paper-based methods (17, 18). This caused non-standardized data collection, thereby complicating the processes of combining, comparing, transmitting and interpreting. As such, RISE conducted data quality audits in 260 health facilities in seventy (70) districts located in five regions to evaluate the quality and reliability of COVID-19 vaccination data. Health facilities were selected using the Dose-Target Coverage (DTC) approach: districts were grouped into vaccination coverage categories of ≥25, ≥50, and ≥75%. Simple random sampling was used to select facilities from each coverage group. Data quality was assessed using the accuracy, completeness, and timeliness dimensions. Source documents were used as gold standard and the Quality of System index (qi) was used to assess data quality. In our findings, we observed that data processing and reporting to higher levels were not accurately coordinated. Further, data documentation was poor with significant deviations in reported data and source documents, prompting regional data quality dissemination across the country.

To address these challenges, RISE trained 120 healthcare providers (including district and regional directors, health information officers, disease control officers, and health promotion officers) across 70 districts in five regions on efficient data reporting and management. RISE also developed and institutionalized standardized reporting tools and implemented monthly data verification processes. Monthly data verification between various reporting levels was conducted and integrated into three (3) vaccination campaigns. These practices, including data quality dissemination significantly improved accurate data capture on vaccination coverage, indicating an increase 93% within three months. By implementing standardized reporting tools at all levels, we achieved data harmonization, which facilitated more effective decision-making. These improvements in data collection standards are not only vital for the COVID-19 pandemic but also for future public health emergencies. Investing in robust data management systems ensures that health policies are based on accurate and reliable data, strengthening the overall resilience and responsiveness of the health system (19, 20). Additionally, reliable data offers options for accountability and transparency, which is critical in building trust among stakeholders, including the public, healthcare providers and donors during pandemic.

### Human resource

Human resources can be defined as all people engaged in actions whose primary intent is to enhance health (11). At early stages of the pandemic in Ghana, 5.1% of patients experienced moderate-to-severe symptoms (21). While the majority of patients were asymptomatic, about 38.9% had underlying non-communicable diseases. Additionally, the severity of infection was primarily influenced by cardiovascular comorbidities, which often required intensive care (21). The RISE project implemented a Basic critical care training course, with an initial aim of dealing with inadequate knowledge on managing critically ill patients. The RISE project collaborated with the GHS and the National COVID-19 Case Management Team to provide a Basic Critical Care course on COVID-19. This modular training adopted a blended learning approach. There were three-week didactic lectures and hands-on training at the Ghana Infectious Disease Center, followed by a week of virtual training. Additionally, the program established a network of critical care consultants accessible via Zoom, WhatsApp, and phone calls, offering tele-consultation to healthcare workers at treatment centers. This assistance included virtual clinical rounds, mentoring, and mental health support. Training modalities included lectures, demonstrations, group discussions, interactive Q&A sessions, practical learning stations, and quizzes. Lecture materials and supplementary electronic textbooks were distributed to participants for future cascade training.

The Basic Critical Care course (BCC) significantly enhanced critical care for COVID-19 patients in Ghana. The course's success highlights its feasibility and lasting impact, suggesting potential for expanding Ghana's health workforce through modest investments in internet and technology access. This form of training has been seen to be useful in improving knowledge, regardless of prior experience and specialty (22). The BCC training has resulted to the recovery of 87.4% of patients admitted to health facilities with a mortality rate of 6.9%; a significant improvement from the 34.4% reported in a major referral hospital prior to the training in Ghana (Supplementary Table 2). This initiative highlights the value of the Basic Critical Care course in bolstering the health workforce during challenging times. Future endeavors in critical care during pandemics should consider adopting similar strategies and scaling up healthcare worker training initiatives. While celebrating this achievement, it's imperative to safeguard and build upon it for continued progress.

It was estimated that 10–15% of COVID-19 patients affected by the Delta and the Omicron variants exhibited acute respiratory insufficiency and required intensive care unit admission to receive advanced respiratory support (23). However, most of the ventilatory and oxygen support techniques require highly trained professionals in Ghana. Further, many health facilities faced several challenges in terms of demand for oxygen therapy, due to the sudden increase in the number of patients requiring oxygen therapy, problems with the medical oxygen supply chain, lack of capacity to deliver oxygen therapy due to infrastructure limitations, and increased competition for resources and staff training. RISE, in collaboration with the Ghana Health Service, provided training to equip health workers strategically in providing oxygen to patients. Overall, 1,549 health workers were trained in 91 health facilities (Supplementary Table 3). This training highlighted improved skills of clinicians in handling oxygen equipment, as well as how to administer oxygen, monitor and maintain oxygen saturation levels. To ultimately strengthen the oxygen support skill in health facilities, we further conducted end-user training for biomedical engineers on the use of oxygen equipment, such as Nidek Max 30, and the operation of PSA plants. This training equipped biomedical engineers with the necessary skills to support clinicians in delivering oxygen to patients. Along with the provision of care, clinicians were also trained in the use of this equipment to harness oxygen delivery.

Moreover, training and capacity-building efforts were often segregated, targeting only clinicians, which resulted in a lack of collaboration and understanding in delivering essential care (2, 24). While clinicians have been at the forefront of providing case management services during the pandemic, engineers have also played a crucial role by offering technical assistance in utilizing various medical equipment and ensuring smooth logistics and supply chain management (25). Ghana's healthcare system has devised innovative strategies to support frontline medical staff during the pandemic, addressing daily challenges and adapting infrastructure as needed. Additionally, educational initiatives swiftly introduced new safety protocols for healthcare providers, minimizing risks in clinical settings (26).

However, the lack of collaborative learning opportunities for clinicians and non-clinicians posed significant challenges, leading to issues like improper equipment usage. To address this, RISE developed an innovative approach – *Joint Clinical and Non-clinical Training*, to facilitate continuous and collaborative learning for both clinicians (physicians, nurses, midwives) and non-clinicians (engineers). Given our resource constraints, this integrated training approach was indispensable for enhancing healthcare delivery. The joint clinical and non-clinical training approach has yielded significant benefits, including enhanced technical and clinical performance among healthcare providers, increased confidence and comfort in their roles, improved collaboration, and expanded participation across various disciplines. This innovative training method has proven to be both beneficial and cost-effective, as it fostered interprofessional learning and provides ample opportunities for strong interpersonal interactions among trainees. Despite the overall success of the training, many challenges were discovered. One major challenge was the initial reluctance of both clinicians and engineers to participate in a collaborative training session. This hesitation arose from conflicting perceptions: clinicians believed that engineers were entirely responsible for equipment maintenance, whereas engineers perceived clinicians as the primary cause of equipment damage owing to inappropriate handling. However, the training's practical, hands-on design encouraged a better knowledge and appreciation of unique roles of clinicians and engineers, emphasising the importance of collaborative capacity building. Furthermore, engineers received additional training on Infection Prevention and Control (IPC), which emerged as a significant issue during the sessions, particularly in dealing with clinicians‘ worries about contamination hazards. Moving forward, we recommend that joint clinical and non-clinical training be adopted to strengthen hospital pandemic preparedness. Furthermore, future research should focus on exploring the long-term impact of this training on healthcare professionals' performance, interdisciplinary collaboration, and patient outcomes.

Studies have recommended that patients with mild symptoms can be considered for home treatment for symptoms, such as fever, sore throat, or cough, while maintaining adequate nutrition and hydration (25, 27). Home-based primary care allows patients to receive long-term medical treatment in their own homes with the enabling support of their families and loved ones (28). Multiple studies have indicated that home-based training for healthcare workers improves person- and caregiver-centered outcomes while saving resources (29). During the pandemic, RISE in collaboration with GHS trained 45 Trainer of Trainees (ToTs) on home-based care who subsequently cascaded the training to 764 health workers (Supplementary Table 4). Our experience with home treatment of low-risk patients has helped maintain limited hospital beds, oxygen supply, and critical care capacity for severe cases, increasing the overall system responsiveness during peak transmission periods.

The national planning for COVID-19 vaccine distribution focused on developing an enabling policy environment, procurement system, and resource allocation. At the district and sub-district levels, the successful introduction, uptake, and equitable distribution of COVID-19 vaccines rely on robust and ongoing operational microplanning. This further requires advocacy and social mobilization work to generate demand to drive people towards uptake. These strategies required effective planning and implementation. To do this, RISE trained frontline health workers on microplanning for COVID-19 vaccination. It entailed creating a detailed plan for deploying COVID-19 vaccines in the catchment area of a primary healthcare center or similar institution. The microplan contains components for managing human resources, vaccines, and logistics; demand generation and communication; service delivery; and community engagement. These efforts were co-created with the GHS, which enabled effective efforts in boosting the integration of routine vaccination and other health services with COVID-19 vaccination as a sustainable and cost-effective option in Ghana. This strategy has contributed to the achieving of 93% vaccination target. Like Nigeria and Somalia, at the community level, healthcare workers used microplanning during the global vaccination campaigns to identify unreached and hard-to-reach communities and make them a priority for future health services. The lessons learned from this intervention laid the foundation for the scale-up of the RISE Global VAX project. Microplanning is an important intervention that enables timely realignment and reprogramming while also offering active assistance to frontline health workers to guarantee effective resource allocation and the development of targeted interventions.

Amid the COVID-19 pandemic, efforts to build capacity primarily targeted healthcare workers on the frontline of COVID-19 care, such as those in treatment centers and emergency units (30). However, units like maternal wards, labor wards, maternity theaters, pediatric units, Special Newborn Care units, and Neonatal Intensive Care Unit (NICU) wards were not considered frontline care areas. Consequently, healthcare workers in these units missed out on essential oxygen training and capacity-building opportunities (31, 32). In response, RISE and GHS conducted an assessment of the oxygen ecosystem, specifically in Maternal, Newborn, and Child Health (MNCH) areas in Ghana. This assessment offered a better understanding of existing training needs related to oxygen provision in these critical healthcare units. Through this initiative, we addressed the overlooked areas of maternal wards, labor wards, maternity theaters, pediatric units, Special Newborn Care units, and Neonatal Intensive Care Unit (NICU) wards, ensuring that healthcare workers in these units received the necessary training to effectively manage oxygen therapy for COVID-19 and other medical emergencies. This led to the training of 215 health workers from 73 health facilities. We recommend that essential health sectors, such as Maternal, Newborn, and Child Health (MNCH), be intentionally prioritized at every level of management in these critical areas. Building a sustainable network of collaboration and establishing continuity, RISE provided training to health workers using trainers who had received earlier training as master trainers.

Patients with underlying disorders had a significantly higher fatality rate than patients without underlying conditions (10.5% for cardiovascular disease, 7.3% for diabetes, 6.3% for chronic respiratory disease, 6.0% for hypertension, 5.6% for cancer, and 0.9% for none). In Ghana, individuals without comorbidities were less likely to die or suffer severe disease from SARS-CoV-2 compared to those with comorbid conditions (33). This necessitates additional attention to be paid to patients who are critically ill, to ensure prompt referrals and escalation of care for deteriorating patients. To address this challenge, RISE conducted training on Acute Hypoxia for clinicians managing critically ill patients beyond COVID-19. In addition, RISE supported basic emergency care training to enhance early recognition and management of deteriorating patients. This approach closely aligns with the learning practice theory described by Billett (37). The training focused on the recognition and care of deteriorating patients.

**Figure 2:**
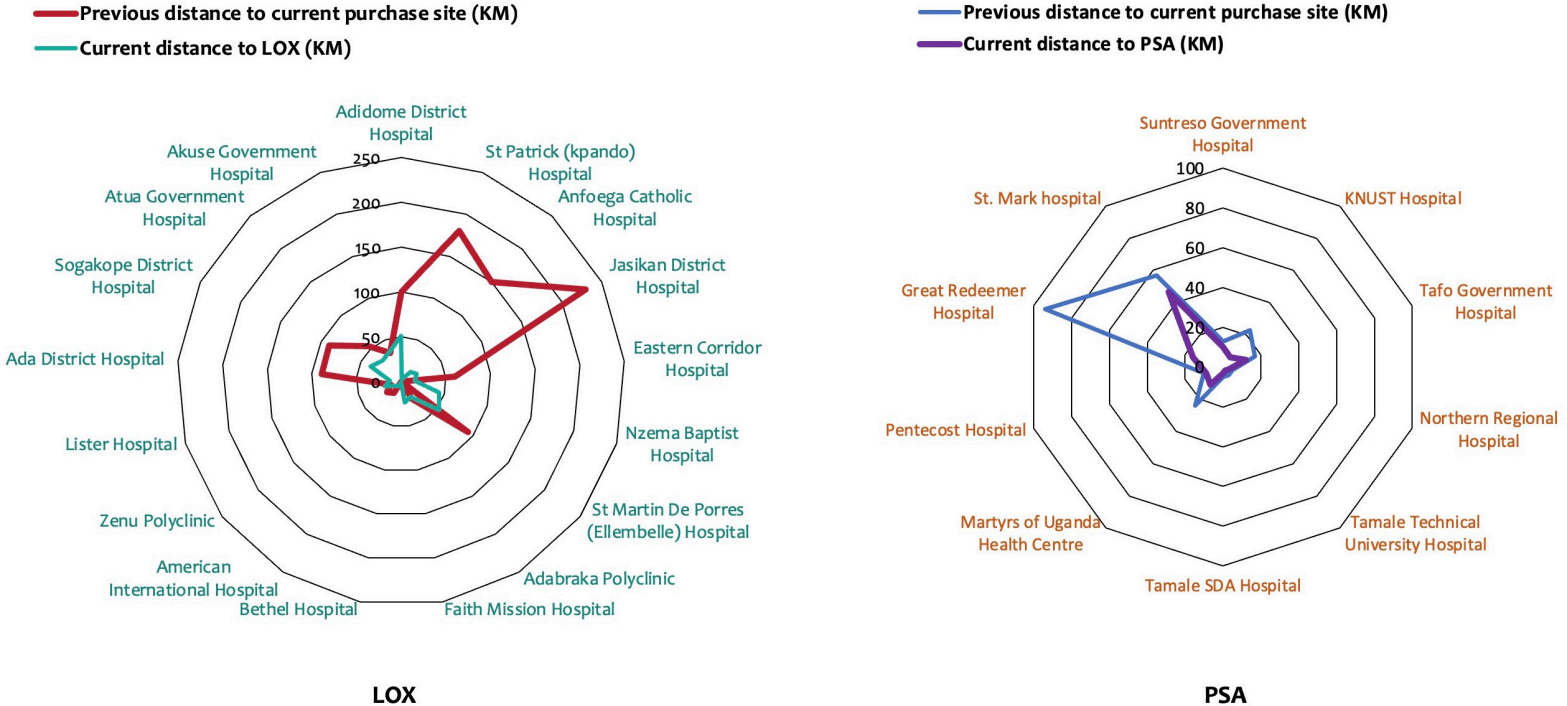
Before and after traveling distance to medical oxygen purchase.

From 2021, RISE with funding with US Government has supported selected hospitals with oxygen infrastructure, including 5 PSA plants, 28 high-flow, high-pressure oxygen concentrators, 10 LOX tanks, and hundreds of oxygen cylinders. Previously, peripheral facilities that relied on LOX travelled distances ranging from 1 km to 230 km to obtain oxygen. Travel distance have decreased to a range of 1 km to 52.4 km since the implementation of LOX systems, with an average distance of 23.2 km compared to 76.7 km previously, a 69.7% reduction. Similarly, facilities located near PSA locations now travel an average of 37 km to access oxygen, a reduction from 51.9 km previously, representing a 12.1% reduction in distance (Figure 2).

To ensure the sustainability of PSA plants, the RISE team, with the Health Administration and Support Services (HASS) of the GHS, supported biomedical engineers to undertake the original equipment manufacturers (OEM) certification training (provided by AirSep) for PSA plants, the LOX cryogenic tanks and dependencies, and the Nidek high-flow high-pressure concentrators. Eleven health workers were trained, comprising 8 from GHS and 3 from Rikair. The GHS team comprised 4 BMEs, 1 from Nsawam Government Hospital, 1 from Tema General Hospital, and 2 from LEKMA Hospital. These pieces of training improved the knowledge and skills of the Ghana Health Service biomedical engineers in providing planned preventive maintenance and corrective maintenance of oxygen infrastructure after warranty and service level agreements (SLAs) on equipment. Further, RISE and GHS-HASS provided cascade training for other biomedical engineers in service, led by the engineers who received the OEM training.

### Health management and leadership

Leadership involve ensuring that strategic policy frameworks exist and are combined with effective oversight, coalition-building, regulation, attention to system design, and accountability (11). One key element of good leadership is accountability, which helps to legitimize increased funding while requiring demonstrable results. During the COVID-19 vaccination campaigns, we required effective coordination at all levels of care in the various regions. To tackle this issue, RISE established an office with clearly defined roles and responsibilities for *Global VAX regional officers*. Accountable health-system governance should be measured against five main criteria: (1) solidifying an understanding of how services are supplied; (2) financing to ensure that adequate resources are available to deliver essential services; (3) performance around the actual supply of services; (4) receipt of relevant information to evaluate or monitor performance; (5) enforcement, such as imposition of sanctions or the provision of rewards for performance (10). When current global health governance is measured against these criteria, some strengths and weaknesses are exposed. These Regional Officers, stationed at regional health directorate offices, served as primary contacts in their respective regions, playing pivotal roles in field supervision, data collection and validation, and addressing challenges while assisting in resource and logistic mobilization. Also, the RISE project played a pivotal role in driving the success of COVID-19 vaccination efforts in Ghana by fostering invaluable collaboration, planning and engagement with the Ministry of Health (MOH), healthcare providers, health managers/Divisions, members of the public/community and the media. These collaborations provided clear road maps for the development and implementation of strategies, health system, government and support and validation. By providing comprehensive training, essential logistics, and effective mobilization strategies, they actively engaged stakeholders at the national, regional, and district levels. These partnerships and coordinated efforts significantly accelerated the progress and impact of the vaccination programs across the country. Aligned with some global recommendations, this initiative significantly contributed to the successful vaccination of millions of individuals, fostering trust for additional funding to support targeted vaccination campaigns.

### Sustainable health financing

Financial Mitigation and plan occur around the mobilization, accumulation, and allocation of money to cover the health needs of the people, individually and collectively (11). The primary goal of health financing is to ensure Universal Health Coverage (UHC) or equitable access to care for all. In Ghana, the main sources of the health financing system include government or donor support, the National Health Insurance Scheme (NHIS), and out-of-pocket payments (34). It is worth noting that the pandemic triggered momentum to develop an alternative yet rapid financing mechanism during this uncertain time in order to increase access to care, if possible, provide healthcare services for free. One of the significant achievements we made in health financing was the operational support we provided to facilitate vaccination mobilization efforts. A performance-based allowance system was effectively established with RISE's direction to incentivise health professionals, ensuring they received direct electronic reimbursements for their participation in these vital activities. This initiative was a collaborative effort with the Ghana Health Service, which played a pivotal role in ensuring that the motivation was directly tied to performance, thereby enhancing efficiency and accountability. Additionally, the project made substantial contributions by providing logistics to support data collection, bolster social mobilization, and improve risk communication. RISE also supplied essential information, education communication (IEC) materials, and infection prevention and control logistics to further strengthen the overall health response. This comprehensive approach not only improved operational outcomes but also demonstrated our commitment to supporting healthcare professionals and enhancing public health infrastructure.

### Service provision

Service provision serves as an immediate output of the inputs into the health system, such as the health workforce, procurement and supplies, and financing (11). There was co-administration of COVID-19 vaccination with other vaccines provided by the Ghana Health Service at fixed, mobile, outreach, or other sites. Over 100,000 persons were vaccinated, excluding pregnant women and children aged 15–17 years. In addition to vaccination service delivery, there was a need to adopt a paradigm shift – from targeted campaigns to routine immunization. RISE conducted an *experimental integration assessment* for COVID-19 vaccination integration into health services. This assessment was geared towards health system strengthening and resilience. The assessment provided valuable insights to the Ghana Health Service (GHS) as they prepare for routine vaccination; collaboration at all levels of care was critical for integration (Supplementary Table 1). To further sustain routine care and management of COVID-19 cases, rapid diagnostic testing and oral antiviral treatment for COVID-19 need to be included in routine care. The Ministry of Health (MoH), Ghana, indicated a strong interest in adopting the test-to-treat (T2T) model for COVID-19. This strategy stems from the need to increase and extend access to testing and reduce delays in treatment. The RISE team supported the T2T implementation by facilitating access to antivirals. The T2T initiative offered a comprehensive health facility-level initiative that provided medications for COVID-19 patients, including rapid tests, to eligible patients at selected health facilities. Under project, 99.9% of suspected patients with COVID-19 were tested; 5.4% tested positive and 71.9% were prescribed oral antivirals within the reporting period of 0 – 5 days. Under this program, eligible persons aged 18 years and older with a current positive COVID-19 test were linked to care. Further, eligible people aged 18 years and older with a current positive COVID-19 test were linked to care.

### Products and logistics

An important goal of a health system is its ability to ensure equitable access to essential medical products, vaccines, and technologies that are safe, effective, and cost-effective (11). The framework emphasizes that a well-functioning health system ensures equitable access to essential medical products, vaccines, and technologies of assured quality, safety, efficacy, cost-effectiveness, and scientifically sound use. Medical oxygen has always been scarce in Ghana, and in early 2020, the country faced a significant shortage. Oxygen cylinders were difficult to find and expensive when available. The arrival of COVID-19 exacerbated this issue, increasing the demand for oxygen as more people experienced respiratory distress. The USG funded the installation of Pressure Swing Adsorption (PSA) plants, which convert ambient air (containing 21% oxygen) into medical-grade oxygen (approximately 90% oxygen) in five hospitals in Ghana. These hospitals serve as hubs for the filling of cylinders for nearby health facilities. RISE has also invested in the installation of 10 liquid oxygen plants and provided high-pressure, high-volume oxygen concentrators to key facilities across all 16 regions. The goal of RISE was to work with the health service to make available multiple generating sources of oxygen in the country to improve access to medical oxygen.

## Discussion

RISE has provided preventive and clinical support in the fight of COVID-19 using novel strategies in Ghana. We see these strategies to be effective, resource responsive and adaptable. Data driven decision making is not only a useful option for patient management but also to aspects of healthcare and the systems around healthcare (35). Our data integration techniques for example – development of standardized data collections, development of real-time dashboards and ensuring data quality were found to be pivotal in the fight against COVID-19. These initiatives were found to be repetitive in Nigeria where the Electronic Management of Immunization Data (EMID) system was optimized to manage COVID-19 vaccination data (36). Concurrently, other cases from Egypt, Kenya, Senegal, Cameroon and South Africa revealed differences in preparedness data (18). Further, effective collaboration with the Ministry of Health, Ghana Health Service and other partners has pivoted both feasibility and sustainability of interventions. Our basic critical care and joint clinical and non-clinical training models for example provided the infrastructure for scale-up to other health facilities. These models reduced silos between service delivery and support functions. The key message of these innovations is targeted towards policy that triggers the use of interoperable health information systems as the basic standard for data collection during pandemics.

To improve ongoing and future emergency planning, interventions should proactively include health system resilience initiatives for essential health services. Specifically, this entails developing frameworks and guidelines with relevant partners in sustaining, coordinating and improving strategies. For RISE activities, several frameworks have been developed to preserve gains, most notably the LOX/PSA sustainability framework, which is intended to ensure that oxygen plants continue to run and operate properly. While this equipment came with warranties and SLAs that covered preventative maintenance and part replacement, these arrangements are time-bound. To address this, the RISE in collaboration with GHS developed guidelines to help create long-term plans for managing oxygen equipment. These principles serve as a template for service delivery points, emphasising coordination, operations, maintenance and inventory management, human resource development and financial sustainability. Additionally, training manuals have been developed to institutionalise and sustain important capacity-building interventions. Recognising the need for strong data systems for informed decision-making, RISE in collaboration with GHS have incorporated data reporting for some key intervention service data (T2T) into the national data platform, the District Health Information Management System (DHIMS2). This initiative projects the importance of such integration, notably in terms of increasing service uptake. This not only reinforced national-level decision-making, but also paved an opportunity for more programmatic integration within national level health system. Furthermore, as of 2024, Ghana was yet to roll out the integration of COVID-19 vaccination into routine health services.

## Conclusion

RISE, the Ministry of Health, and the Ghana Health Service took strategic steps to mitigate the effects of the pandemic in Ghana. Other partners also played a similar role, working collaboratively with the government. Responding to the COVID-19 pandemic using an integrated health systems strengthening approach enhanced global health security, enabling better control of the pandemic. We urge public health policymakers, partners, and donors to fully support and sustain these interventions to build a more resilient health system.

## Data Availability

The original contributions presented in the study are included in the article/Supplementary material, further inquiries can be directed to the corresponding author.
