# Supplementary material for: COVID-19 in Ghana-Using the health system framework to describe RISE-mitigation strategies for COVID-19 response and recovery in Ghana
Source: Front Public Health. 2026 May 25;14:1810964. doi: 10.3389/fpubh.2026.1810964 (PMC13243293; doi:10.3389/fpubh.2026.1810964)
Supplement: Supplementary file 1 [file Data_Sheet_1.pdf]

Supplementary File

Table of Contents

Supplementary Table 1: Criteria for mapping projects to building blocks ... 3
Supplementary Table 2: Cases seen by facilities and their outcomes as at the time of visit ... 3
Supplementary Table 3: Summary of oxygen therapy training ... 4
Supplementary Table 4: Case management trainings data summary ... 5
Supplementary Table 5: Types of stakeholders involved in prioritizing guideline topics and the methods of engagement ... 6
Supplementary Table 6: Key findings RISE experimental introduction of COVID-19 vaccination integration into health services in five regions of operation ... 8
Supplementary Table 7: Summary of T2T service delivery ... 10
Supplementary Table 8: Descriptive of RISE-led interventions during the COVID-19 wave 11

Supplementary Table 1: Criteria for mapping projects to building blocks

Budling Block	Criteria
1. Information Systems and Data Integration	Included projects that involved digital technologies, electronic health records, survey and data collection systems, dashboards and system interoperability
2. Human Resource	Projects that focused on capacity building/training
3. Health Management and Leadership	Projects that strengthened management, governance
4. Sustainable Health Financing	Project that improves funding mechanisms,
5. Service Provision	Projects that directly improve health service delivery, clinical care, outreach programs and vaccination
6. Products and Logistics	Projects that focused on the procurement of medical equipment and items

Supplementary Table 2: Cases seen by facilities and their outcomes as at the time of visit

Region:	Name of facility:	Cumulative cases recorded	Total cases recovered	Total Deaths recorded	Total active cases at the time
Central	Trauma and Specialist Hospital Winneba	44	35	6	0
Western North	Sefwi-wiawso municipal hospital	138	127	2	9
Western North	Bibiani Government Hospital	269	268	0	1
Ahafo	Goaso Government Hospital	83	76	7	0
Northern	Tamale Central Hospital	20	18	2	0
Ahafo	Techimantia Government Hospital	13	13	0	0
Upper East	Bolga Regional Hospital	490	472	28	0
Central	Cape Coast Metropolitan Hospital	187	181	2	3
Eastern	Koforidua Regional Hospital	602	517	62	23
Bono	Bono Regional Hospital	146	126	20	0
Savannah	Bole District Hospital	0	0	0	0
Upper West	Upper West Regional Hospital	91	78	13	0
Volta	Hohoe Municipal Hospital	478	275	3	3
Bono East	Kintampo Municipal Hospital	142	140	0	2
Western	Effia Nkwanta	544	529	15	0
Oti	Worawora Government Hospital	50	49	1	0
Bono East	Holy Family Hospital	256	246	10	0
North East	Walewale Municipal Hospital	1	0	1	0
Oti	Jasikan Health Centre	52	52	0	0
Oti	Nkwanta South Municipal Hospital	79	79	0	0
Ashanti	Kumasi South Hospital	260	239	21	6
Greater Accra	Ridge Hospital	324	210	103	11
**Total**		**4,269**	**3,730 (87.4%)**	**296 (6.9%)**	**58 (1.3)**

Supplementary Table 3: Summary of oxygen therapy training

	Cumulative number of health workers trained 2022 - 2023									
	**Prior to July**	**July**	**August**	**September**	**October**	**November**	**December**	**January**	**February**	**March**
TOTAL number of facilities receiving oxygen clinical technical assistance training	0	0	0	16	38	45	59	59	74	91
TOTAL number of health workers that received oxygen clinical technical assistance training	0	0	0	60	142	196	255	255	331	486
Female	0	0	0	16	50	63	83	83	109	197
Male	0	0	0	44	92	133	172	172	222	289
Unknown/No gender data available	0	0	0	0	0	0	0	0	0	0
By Cadre	0	0	0	0	0	0	0	0	0	0
Physician	0	0	0	11	37	49	62	62	84	103
Nurse	0	0	0	46	84	109	135	135	171	240
Clinical Officers/ Physician Assistants	0	0	0	2	4	16	18	18	35	44
Respiratory therapists (RT) or equivalent	0	0	0	0	0	0	0	0	0	6
Physical therapist (PT) or equivalent	0	0	0	0	0	0	0	0	0	2
Pharmacists	0	0	0	0	0	0	0	0	0	1
Other	0	0	0	1	17	22	40	40	41	90
**Total**	**0**	**0**	**0**	**196**	**464**	**633**	**824**	**824**	**1067**	**1549**

Supplementary Table 4: Case management trainings data summary

No	Region	Case Mgt (ToT HBC Training)	Case Mgt (HBC cascade Training)
1	Ashanti	4	81
2	Ahafo	3	38
3	Bono	4	49
4	Bono East	2	42
5	Central	3	49
6	Eastern	0	61
7	Greater Accra	6	84
8	Northern	4	64
9	North East	1	28
10	Oti	2	33
11	Savannah	3	40
12	Volta	4	50
13	Upper East	3	32
14	Upper West	3	36
15	Western	3	49
16	Western North	0	28
**Total**		**45**	**764**

Note: Regions with zero ToT (Western North and Eastern) had relocation of other regional ToTs due to staff attrition

Supplementary Table 5: Types of stakeholders involved in prioritizing guideline topics and the methods of engagement

		Ministry of Health	Ghana Health Service (GHS)		Public and Community Actors		Academic	Other Partners	
		Ministry	Healthcare providers	Health managers/ Divisions/ Departments	Members of the public/ community	Media	University/ Researchers	Non-governmental organizations	Overall
**Financial mitigation and Plan**									
	Vaccination mobilization Performance-linked allowance system		✓	✓	✓	✓			
	Essential IEC materials support					✓			4
**Health management and leadership**									
	Regional officer integration into the R/DHDs Office initiative		✓	✓					0
**Products and logistics**									
	LOX	✓	✓	✓	✓			✓	0
	PSA	✓	✓	✓				✓	5
**Information Systems and Data Integration**									
	Saturation analysis		✓	✓	✓				0
	Data quality audits (DQA)		✓	✓					3
	Power BI dashboard								2
**Human Resource**									
	Joint clinical and non-clinical training	✓	✓	✓			✓		0
	Basic Critical Care (BCC) training	✓	✓	✓			✓		4
	MNCH oxygen training		✓	✓					4
	OEM training for biomedical engineers		✓	✓			✓	✓	2
	Basic emergency care training		✓	✓			✓		4
	Micro-planning		✓	✓					3
	Training on Acute Hypoxia		✓	✓					2
	Home-based care training		✓	✓					2
	End-user training for biomedical engineers		✓	✓				✓	2
	Training on oxygen therapy		✓	✓					3
**Service provision**									
	Global vaccination (GVAX)	✓	✓	✓	✓				0

	Test-to-Treat	✓	✓	✓	✓	✓			4
	Experimental integration		✓	✓					5
**Total**		**6**	**19**	**19**	**5**	**3**	**4**	**4**	**60**

Note:

Ministry: Throughout the implementation of the Global vaccination (GVAX) and Test-to-Treat interventions, the Ministry of Health (MoH) Ghana provided oversight of country level approval of treatment guidelines and protocols of vaccines and medications. Additionally, MoH, spearheaded policy oversight and regulations that effectively coordinated all relevant partners (UNICEF, GAVI).

Healthcare providers: Healthcare providers were direct stakeholders of capacity building interventions. Through strategic application of inclusion criteria, health workers were selected to participate in training modules, cascade trainings (online-offline).

University/Researchers: The scientific community provided structured module courses for the training of health workers. These modules were validated and approved by GHS

Members of the public/Community: Participated in surveys and community engagement activities

Media: Provided wider and national coverage of the T2T project through media adverts on Covid-19 medication

Health managers: Provided onsite, district, regional and national level supervision of all interventions through joint project management (JPM) initiatives.

Non-governmental organizations: Partnered with other stakeholders (USAID-PSM) on MNCH assessment, RIKAIR for liquid

RISE
Reaching Impact, Saturation and Epidemic Control

Supplementary Table 6: Key findings RISE experimental introduction of COVID-19 vaccination integration into health services in five regions of operation

	Assessment Criteria	Key Findings
1	Leadership and governance	**RHD**<br>-Coordinated engagement meetings between RISE and managers of the various districts and facilities to plan for the integration.<br>-Supported monitoring and supervision of integration activities at the facilities.<br>-Facilitated logistical support (COVID-19 vaccine, syringes/needles, recording and reporting tools) to districts and facilities for integration<br><br>**DHD**<br>-Facilitate engagement of organized social groups and support other demand generation efforts by facilities.<br>-Support resources and logistics mobilization for vaccination integration (personnel, COVID-19 vaccine, syringes/needles, cotton, vehicle, data capture tools, etc.)<br><br>**Health Facility**<br>-Appointed physicians as leads for integration activities in the facility.<br>-Sensitized hospital management teams, Heads of specialized clinics/other unit In-charges and physicians on the integration<br>-Lead the formation, orientation and setting up of vaccination teams at the specialized clinics and other relevant units within the facility.<br>-Ensure supply of ancillary logistics such as cotton wool, emergency drugs etc.<br>-Lead monitoring and supervision of facility-based vaccination integration
2	Country Policy /Guidelines on immunization	The assessment revealed that the approach to integration was in line with relevant country EPI policy and guidelines. Key policy areas mentioned by respondents that aligned with the integration were:<br>- Adherence to existing vaccine handling, administration and safety monitoring guidelines.<br>- Data capture and reporting tools are in line with data management protocols of GHS.<br>- Orientation was done for vaccination teams prior to integration which aligns with Training/Capacity building as aspect of policy guidelines.<br>- Linkage with essential services fits well with service delivery integration by the GHS for optimum use of resources.<br>- The respondents also expressed their satisfaction that RISE followed the command structure of the GHS to introduce the integration of Covid-19 vaccination. It allowed them to own the process.
3	Financing of COVID-19 vaccination integration	The assessment revealed various other ways how other logistics such as (cotton wool, zip-lock bags, sanitizers, Emergency drug, A4 paper) were obtained for the integration activities. The approaches include the following:<br>- Ancillary logistics were provided from routine supplies from stores of most health facilities.<br>- A few facilities/districts indicated they bought from the open market with their Internally Generated Fund (IGF) and part of the frontload funds from RISE for the targeted campaign.<br>- A total of 26 out of 48 facilities visited indicated they had plans & budgets for integration but however could not produce copies for observation
4	Demand generation	The assessment checked on the effective ways the facilities promoted Covid-19 vaccination within the hospital environment, since hitherto vaccination was community based. Below are some innovative ways deployed by various facilities.<br>- Liaised with consulting room clinicians to do one-on-one counselling of clients, this turn out to be very effective because of the trust clients have for their doctors. This improved acceptance of the vaccine.

		- Sensitization of staff at facility clinical meetings ensured their corporation and support.<br>- Mass education at waiting areas at specialized clinics (HPT, ART, Diabetic, TB) and Outpatient departments<br>- Education at facility's information desk using public address systems<br>- Education of organized groups (Pensioners, artisans, market women, seamstresses/tailors, and hairdressers) during their scheduled meetings.
5	Service delivery	COVID-19 vaccination was integrated with varied combination of routine services at the various health facilities: the routine services included; ART, HPT, TB, Diabetic, CWC, OPD, Physiotherapy, eye clinic, ENT, Family planning, adolescent friendly services, psychiatric service, outreach services, wellness clinics, among other areas.
6	Health Workforce	- Multiple cadres of staff were involved in the integration activities. These included CHNs, DCO, HPOs, PHNs, HIOs, Nurse managers, Administrators, Medical Officers, and General clinical Nurses.<br>- All staff were given some orientation including their roles prior to the integration.<br>- Most facilities (65%) indicated there was no change in workload despite the integration of the COVID-19 vaccination. However, 35% said there was a change in workload siting inadequate immunization staff (CHNs, RCNs, DCOs) coupled with the lack of trained staff on immunization at specialized clinics. As a result, the few available staff had to take up additional duty of being assigned to the specialized clinics for COVID-19 vaccination during the integration.
7	Health information	The data capture and reporting of Covid-19 vaccination followed what has been incorporated in the DHIMS-2 data base by Ghana Health Service health information management system for future roll-out of Covid-19 vaccination integration. However, the respondents indicated the modification in the data capturing tool with additional indicators, which enabled them to categorize the clients by point of vaccination and type of comorbidity was valuable.<br>Existing AEFI surveillance system was used for reporting and managing all COVID-19 related Adverse Events Following Immunization (AEFI).
8	Vaccine and Cold-chain system	- Most facilities indicated the existing cold-chain storage capacity was adequate to accommodate COVID-19 vaccine for the integration. This due to the fact that some adjustments were made to cold rooms at the onset of the pandemic by the GHS and donations of cold equipment from various NGOs and Agencies (WHO, JICA, UNICEF RISE).<br>- A few facilities at the lower levels of service delivery (3 out of 23 facilities visited) complained of either the lack of vaccine refrigerator or faulty vaccine refrigerators.<br>- Most facilities and districts obtain COVID-19 vaccine from the next level through manual request with requisition books. Requisition of Covid-19 vaccine through Ghana Integrated Logistics Management Information System (GiLMIS) and Last Mile Distribution (LMD) like other vaccines is yet be operational.<br>- Majority of the districts and facilities stated the existing waste management system was adequate to manage the additional COVID-19 vaccination related waste and therefore no need for expansion.<br>- A few facilities indicated they made some changes to their existing waste management system to effectively accommodate COVID-19 vaccination related waste, the changes included; 1. Increase in quantity of fuel for incineration, 2. increased personnel required to do waste management.

Supplementary Table 7: Summary of T2T service delivery

	Total	Age group			
		<15	15-49	50+	Unknown
Total number of patients with suspected COVID-19 illness	7348	329	4876	2066	77
Number of patients suspected of COVID-19 who were tested	7345	329	4874	2065	77
Total number of patients confirmed to have COVID-19 illness	395	13	283	96	3
Total number of patients prescribed oral antivirals within the reporting period	284	5	214	62	3
	Total	**Time from symptom onset to uptake of care**			Total
		0-5 days	6+	Unknown	
Total number of patients with suspected COVID-19 illness	7348	3591	2557	1200	7348
Number of patients suspected of COVID-19 who were tested	7345	3590	2557	1198	7345
Total number of patients confirmed to have COVID-19 illness	395	264	85	46	395
Total number of patients prescribed oral antivirals within the reporting period	284	208	40	36	284
	Total	**Presence of additional high-risk criteria**			Total
		Yes	No	Unknown	
Total number of patients with suspected COVID-19 illness	7348	1398	4888	1062	7348
Number of patients suspected of COVID-19 who were tested	7345	1397	4887	1061	7345
Total number of patients confirmed to have COVID-19 illness	395	112	243	40	395
Total number of patients prescribed oral antivirals within the reporting period	284	74	187	23	284
	Total	**Symptom Severity**			
		Asymptomatic	Mild to Moderate	Severe or Critical	Unknown
Total number of patients with suspected COVID-19 illness	7348	1373	4315	127	1533
Number of patients suspected of COVID-19 who were tested	7345	1373	4315	127	1530
Total number of patients confirmed to have COVID-19 illness	395	48	261	14	72
Total number of patients prescribed oral antivirals within the reporting period	284	40	183	3	58
	Total	**Recieved at least first dose of primary vaccine series**			Total
		Yes	No	Unknown	
Total number of patients with suspected COVID-19 illness	7348	3981	832	2535	7348
Number of patients suspected of COVID-19 who were tested	7345	3979	832	2534	7345
Total number of patients confirmed to have COVID-19 illness	395	235	52	108	395
Total number of patients prescribed oral antivirals within the reporting period	284	182	30	72	284

Supplementary Table 8: Descriptive of RISE-led interventions during the COVID-19 wave

	Description of RSIE-led Intervention	Description of intervention
**Health Management and leadership**		
	Regional Officer integration	This office was established within the Regional and District Health Directorates (RHDs/DHDs) in RISE-implementing regions to provide structured technical assistance for the RISE Global Vax project. Regional officers played a central role in offering technical guidance and oversight in the implementation and management of project activities across regional, district, and sub-district levels.
**Prodcuts and Logistics**		
	PSA	Jhpiego Ghana with collaboration with Ghana Health Service supported 5 facilities with 5 Pressure Swing Adsorption (PSA).
	LOX	To respond to the urgency, and to build resilience for future waves of COVID-19, RISE with collaboration with Ghana Health Service built upon existing gains to expand and improve the oxygen ecosystem in Ghana by procuring and installing liquified oxygen (LOX) as a viable complementary source of medical oxygen for clinical service delivery in 10 hospitals.
**Service Provision**		
	Global Covid-19 vaccination	RISE, in collaboration with the Ministry of Health Ghana and the Ghana Health Service, successfully vaccinated 93% of the 7,540,000 individuals targeted for COVID-19 vaccination.
	Test-to-Treat	Under the Test-to-Treat project RISE in collaboration with Ministry of Health, Ghana Health Service, supported 20 facilities with COVID-19 medications (antiviral) to patients tested positive for COVID-19 within 0 – 5 days.
	Experimental integration	In February 2024, Ghana was yet to roll out Integration of COVID-19 vaccination into health services, though guideline for integration was ready it was yet to be disseminated to the health care workers. RISE conducted an experimental assessment on the introduction of COVID-19 vaccination integration into routine health services in five regions. This assessment provided lessons which was shared with the Ghana Health Service.
**Information Systems and Data Integration**		
	Saturation analysis	In Ghana, an ambitious COVID-19 vaccination target was challenged by widespread hesitancy, with only 56.7% of the target population fully vaccinated by 31 December 2023. Despite substantial investments in multiple vaccination campaigns, there remained a critical need to better understand population-level dynamics influencing uptake. In response, RISE, in collaboration with the Ghana Health Service, conducted a large-scale survey across 27,222 households involving 85,106 participants to identify community-level barriers to vaccination, determine preferred vaccination days, and estimate the proportion of individuals willing to be vaccinated.
	Data quality audits	COVID-19 vaccination efforts have generated and reported data over a period of approximately two years, primarily through electronic systems. However, these systems encountered operational challenges,

		leading to the ad hoc use of paper-based methods. In the absence of standardized reporting forms or registers, regions and districts relied on multiple, inconsistent reporting channels. In response, RISE, in collaboration with the Ghana Health Service, developed standardized reporting tools and delivered targeted training for health workers and managers on data validation and reporting. This initiative significantly improved data quality and strengthened the reliability of information available for decision-making.
	Power BI dashboard	Vaccination campaigns experienced high levels of refusal, necessitating an assessment of refusal patterns by geographical location to better inform campaign implementation and resource allocation. This required the application of spatial analytical approaches, as traditional methods often do not capture spatial clustering and distribution patterns, and existing tools lack the capacity to detect statistically significant hot and cold spots. In response, RISE developed a dashboard that integrates spatial techniques including aggregation analysis, kernel density estimation (KDE), and Getis-Ord Gi* hotspot analysis to identify and visualize significant clusters of COVID-19 vaccination refusals, while also providing real-time vaccination data across Ghana.
**Sustainable Health Financing**		
	Vaccination mobilization performance-linked allowance system	A performance-based allowance system was established under RISE's guidance to incentivize health professionals, with direct electronic reimbursements provided for their participation in these critical activities. Implemented in collaboration with the Ghana Health Service, the initiative ensured that incentives were closely linked to performance, thereby improving efficiency and strengthening accountability.
	Essential IEC material support	RISE in collaboration with Ghana Health Service (Health Promotion Division) developed information, education communication (IEC) materials to support uptake of health services (COVID-19 vaccination and medications under the T2T project)
**Human Resource**		
	Micro-planning	To ensure equitable vaccination coverage, it is essential that campaigns are strategically targeted using appropriate approaches and resources. In response, RISE, in collaboration with the Ghana Health Service, implemented microplanning for vaccination campaigns at the district and sub-district levels. Extending microplanning to the community and household levels enhanced inclusivity and reduced missed opportunities for vaccination, both at health facilities and during outreach activities across communities and other service delivery points.
	Joint clinical and non-clinical training	The lack of collaborative learning opportunities for clinicians and non-clinicians (engineers) posed significant challenge during the pandemic. RISE and Ghana Health Service developed an innovative approach, Joint Clinical and Non-clinical Training to promote continuous, collaborative learning between clinicians (physicians, nurses, and midwives) and non-clinicians (such as engineers). In the context of limited resources, this integrated training model proved essential for strengthening healthcare delivery. It fostered interprofessional learning and provided ample opportunities for strong interpersonal interactions among health workers.

	BCC training	RISE and Ghana Health Service, working with the Ministry of Health (MoH) and the National COVID-19 Case Management Team (NCMT) introduced Ghana's first-ever basic critical care course for health workers managing Covid-19 treatment centers in all 16 regions. This modular training was aimed at equipping health workers with the knowledge and skills to manage patients with COVID-19, especially those with severe and critical illness. This basic critical care course was a four-week modular training using blended learning techniques – involving in-person didactic and hands-on skills training component, and a virtual training component. The training focused on assisting the trainees in setting up a high-dependency unit when the candidates returned to their home facilities. The modular training built a community of practice of health workers with intensive care specialists, infectious disease specialists, and other consultants who were readily available on Zoom, WhatsApp, or voice calls to provide tele-support at treatment centers.
	MNCH training	Despite ongoing efforts to accurately estimate oxygen demand, insufficient attention has been given to Maternal, Newborn, and Child Health (MNCH) units. A comprehensive assessment within MNCH further identified critical gaps across 73 health facilities involving 215 health workers. These findings underscore the need to deliberately prioritize essential service areas such as MNCH at all levels of health system management. To promote sustainability and continuity, RISE adopted a cascading training model, equipping health workers through trainers who had previously been developed as master trainers, thereby strengthening a collaborative and resilient capacity-building network.
	OEM training	Original Equipment manufacturer (OEM) training on the LOX system* As part of the LOX installation, Auguste Cryogenic, an OEM from Slovakia, conducted a two-day training session on October 8-9, 2025, at LEKMA Hospital in Ghana. The purpose was to enhance the technical capacity of BMEs from the GHS and Rikair regarding the LOX system. A total of 11 participants took part in the training, comprising 8 from GHS and 3 from Rikair. The training covered the LOX system's structural components, filling process, routine maintenance and safety procedures, and operation of the telemetry device used to monitor system operation.
	Basic emergency care training and Training on Acute Hypoxia	In Ghana, individuals without comorbidities were less likely to experience severe disease or mortality from SARS-CoV-2 compared to those with underlying conditions. This requires the need for heightened attention to critically ill patients, including timely referral and appropriate escalation of care for those whose conditions are deteriorating. In response, RISE and Ghana Health Service provided basic emergency care and hypoxia training to strengthen the early recognition and management of at-risk patients.
	Training on oxygen therapy	Home-based primary care enables patients to receive long-term medical treatment in their own homes, supported by their families and loved ones (28). Evidence from multiple studies shows that training healthcare workers in home-based care improves both patient- and caregiver-centered outcomes while also optimizing resource use (29). During the pandemic, RISE, in collaboration with the Ghana Health Service, trained 45 Trainers of Trainers (ToTs) in home-based care, who subsequently cascaded the training to 764 health workers.

RISE
Reaching Impact, Saturation
and Epidemic Control
